# Prediction of Proximal Junctional Kyphosis After Posterior Scoliosis Surgery With Machine Learning in the Lenke 5 Adolescent Idiopathic Scoliosis Patient

**DOI:** 10.3389/fbioe.2020.559387

**Published:** 2020-10-06

**Authors:** Li Peng, Lan Lan, Peng Xiu, Guangming Zhang, Bowen Hu, Xi Yang, Yueming Song, Xiaoyan Yang, Yonghong Gu, Rui Yang, Xiaobo Zhou

**Affiliations:** ^1^West China Biomedical Big Data Center, West China Hospital/West China School of Medicine, Sichuan University, Chengdu, China; ^2^Department of Orthopedic Surgery, West China Hospital, Sichuan University, Chengdu, China; ^3^Department of Ultrasound, West China Hospital, Sichuan University, Chengdu, China; ^4^Center for Computational Systems Medicine, School of Biomedical Informatics, University of Texas Health Science Center at Houston, Houston, TX, United States

**Keywords:** spinal deformity, proximal junctional kyphosis, sagittal malalignment, machine learning, prediction model

## Abstract

**Objective:**

To build a model for proximal junctional kyphosis (PJK) prognostication in Lenke 5 adolescent idiopathic scoliosis (AIS) patients undergoing long posterior instrumentation and fusion surgery by machine learning and analyze the risk factors for PJK.

**Materials and Methods:**

In total, 44 AIS patients (female/male: 34/10; PJK/non-PJK: 34/10) who met the inclusion criteria between January 2013 and December 2018 were retrospectively recruited from West China Hospital. Thirty-seven clinical and radiological features were acquired by two independent investigators. Univariate analyses between PJK and non-PJK groups were carried out. Twelve models were built by using four types of machine learning algorithms in conjunction with two oversampling methods [the synthetic minority technique (SMOTE) and random oversampling]. Area under the receiver operating characteristic curve (AUC) was used for model discrimination, and the clinical utility was evaluated by using F1 score and accuracy. The risk factors were simultaneously analyzed by a Cox regression and machine learning.

**Results:**

Statistical differences between PJK and non-PJK groups were as follows: gender (*p* = 0.001), preoperative factors [thoracic kyphosis (*p* = 0.03), T1 slope angle (T1S, *p* = 0.078)], and postoperative factors [T1S (*p* = 0.097), proximal junctional angle (*p* = 0.003), upper instrumented vertebra (UIV) – UIV + 1 (*p* = 0.001)]. Random forest using SMOTE achieved the best prediction performance with AUC = 0.944, accuracy = 0.909, and F1 score = 0.667 on independent testing dataset. Cox model revealed that male gender and larger preoperative T1S were independent prognostic factors of PJK (odds ratio = 10.701 and 57.074, respectively). Gender was also at the first place in the importance ranking of the model with best performance.

**Conclusion:**

The random forest using SMOTE model has the great value for predicting the individual risk of developing PJK after long instrumentation and fusion surgery in Lenke 5 AIS patients. Moreover, the combination of the outcomes of a Cox model and the feature ranking extracted by machine learning is more valuable than any one alone, especially in the interpretation of risk factors.

## Introduction

For adolescent idiopathic scoliosis (AIS) patients, orthopedic operations are employed to reconstruct the coronal and sagittal alignment in an attempt to maintain the stability of the spine (Mimura et al., 2017). Long posterior instrumentation and fusion surgery is the preferred treatment strategy for improving the management of progressive scoliotic spines (Suk et al., 1995). Although all the efforts have been made to design a suitable operative procedure, the prognosis is not always satisfactory (Humke et al., 1995; Bridwell, 1997). Proximal junctional kyphosis (PJK), a multifactorial proximal adjacent segment disease following fusion treatment, has drawn the attention of many spine surgeons (Watanabe et al., 2010; Kim et al., 2013). It affects around 28% of the adolescent idiopathic scoliosis (AIS) population, with regional pain and poor life quality in some severe cases (Kim et al., 2007; O’Shaughnessy et al., 2012; Passias et al., 2018; Sebaaly et al., 2018). The most commonly adopted definition of PJK is accepted in this study: the Cobb angle between the upper instrumented vertebra (UIV) and the two supra-adjacent vertebrae is superior to 10° and at least 10° greater than its preoperative value (Glattes et al., 2005).

Currently, most researchers are devoted to extracting proper prognostic information by using statistical methods to have an insight into the characteristics with high risks (Kim et al., 2008; Scheer et al., 2016). Previous studies also showed the potential of binary logistic regression in risk factors identification, such as old age, gender, fusion levels, type of instrumentation at the UIV, and various sagittal spinopelvic radiographic parameters (Sebaaly et al., 2018; Zhao et al., 2018). To our knowledge, no reported studies analyzed cervical balance parameters in conjunction with well-known clinical prognostic factors to confirm that it is an independent risk factor for AIS patients. In addition, logistic regression models depend heavily on the linear separability of samples, which is vulnerable to the degree of multicollinearity between variables and may result in a model with underfitting and low accuracy to provide unreliable outcome prediction for a personalized surgical planning. Therefore, it seems unreasonable to make use of linear models for accurate preoperative prediction in the era of personalization of medicine. Non-linear machine learning methods (e.g., random forest) have a distinct advantage over the linear approach because they distinctly provide inherent data pattern recognition and map non-linear relationships between high-dimensional variables to estimate the clinical outcome for each individual (Karhade et al., 2019). Scheer et al. (2016) have constructed a decision tree model (accuracy = 0.860) on 510 adult spinal deformity patients by commercially available software. Nonetheless, in the study, just 13 variables were considered for the highly heterogeneous study population.

The purpose of this study was to establish preoperative risk models for Lenke 5 AIS patients undergoing long posterior instrumentation and fusion surgery. We also explored and compared the outcomes of machine learning and a commonly used model in clinic (Cox regression) at risk factor identification for PJK.

## Materials and Methods

### Patient Population

**FIGURE 1:**
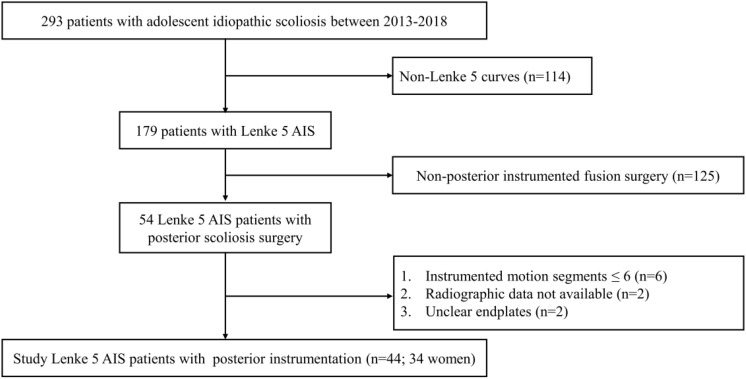
Flow diagram of patient inclusion and exclusion. AIS, adolescent idiopathic scoliosis.

The institutional review boards approved this retrospective study and waived the requirement to obtain written informed consent. Between January 2013 and December 2018, 293 AIS patients were admitted to West China Hospital. Inclusion criteria were as follows: (1) Lenke 5 curves (2) long posterior instrumentation and fusion surgery with > 6 instrumented motion segments, (3) at least 1 year follow-up; (4) adequate preoperative, immediate postoperative (3–7 days after surgery), and final follow-up anteroposterior and lateral standing long-cassette radiographs; (5) radiographs with good quality. Finally, a total of 44 Lenke 5 patients with posterior instrumentation (34 without PJK and 10 with PJK) were recruited on the basis of the eligibility criteria (Figure 1).

### Parameters Collection

Patient demographics and surgical factors including amount of correction, upper instrumented vertebra (UIV) level, lower instrumented vertebra (LIV) level, and the number of instrumented vertebras were recorded from the electronic medical records.

Two coronal and 28 sagittal parameters were collected according to the results of previous researches on PJK (Glattes et al., 2005; Kim et al., 2005, 2013, 2014; Yagi et al., 2011; Hostin et al., 2013; Ghailane et al., 2017; Sebaaly et al., 2018; Zhao et al., 2018; Alzakri et al., 2019). Specifically, coronal parameters included the following: coronal vertical axis (CVA, offset of C7 plumb-line relative to the center sacral vertical line) and the main scoliosis curve Cobb angle (CAMSC); sagittal parameters included the following: the sagittal vertical axis (SVA, offset of C7 plumb-line relative to S1 on the sagittal plane), pelvic tilt (PT), pelvic incidence (PI), PI-LL mismatch, sacral slope (SS), upper segmental lumbar lordosis from L1 to L4 (ULL), lower segmental lumbar lordosis from L4 to S1 (LLL), lumbar lordosis (LL, Cobb angle between superior endplate of L1 and superior endplate of S1), thoracic kyphosis (TK, Cobb angle between superior endplate of T4 and inferior endplate of T12), rod contour angle (RCA, angle between the superior plate of UIV and the inferior plate of one vertebra caudal to the UIV), UIV – UIV + 1 (angle between the inferior endplate of UIV and the superior endplate of one cephalad vertebrae), proximal junctional angle (PJA, angle between the inferior endplate of UIV and the superior endplate of two cephalad vertebrae), T1 slope (T1S, Cobb angle between a horizontal line and the upper endplate of T1), and T1SpinoPelvic inclination (T1SPI, the angle between the vertical plumb-line and the line drawn from vertebral body center of T1 and the center of the bicoxofemoral axis).

**FIGURE 2:**
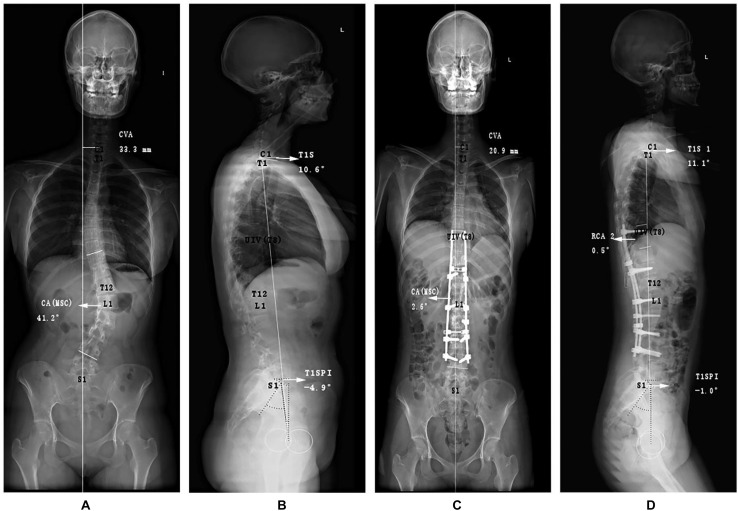
Graphic representations of special angles of an adolescent idiopathic scoliosis patient with PJK postoperatively. Different from the conventional measurements, the **(A)** anteroposterior and **(B)** lateral preoperative radiographs purposely included the following measurements for demonstrating coronal and sagittal malalignment: coronal vertical axis (CVA), the main curve coronal angle (CAMSC), T1 Slope (T1S), and T1SpinoPelvic inclination (T1SPI). At immediate postoperative X-ray films **(C,D)**, rod curve angle (RCA) was also measured. PJK, proximal junctional kyphosis.

**FIGURE 3:**
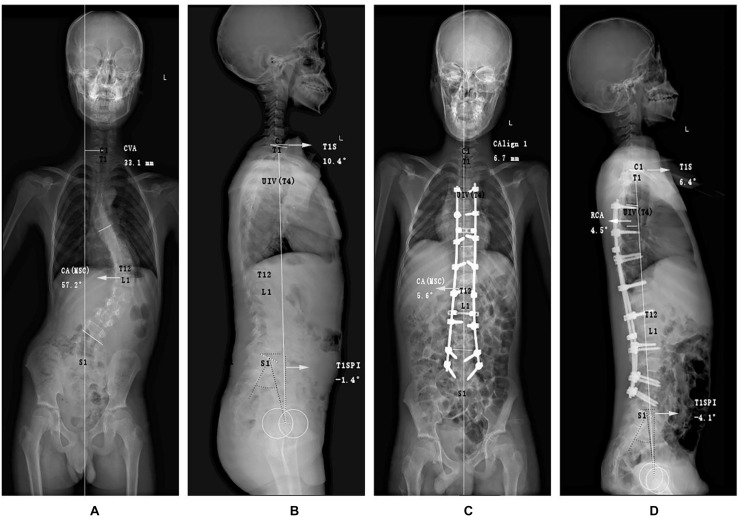
Graphic representations of special angles of an adolescent idiopathic scoliosis patient without PJK postoperatively. Different from the conventional measurements, the **(A)** anteroposterior and **(B)** lateral preoperative radiographs purposely included the following measurements for demonstrating coronal and sagittal malalignment: coronal vertical axis (CVA), the main curve coronal angle (CAMSC), T1 Slope (T1S), and T1SpinoPelvic inclination (T1SPI). At immediate postoperative X-ray films **(C,D)**, rod curve angle (RCA) was also measured. PJK, proximal junctional kyphosis.

It is worthy of note that the value of PI was constant before and after surgery; thus, we only demanded the preoperative PI. Moreover, RCA was defined as a postoperative variable as stated by Kim et al. (2007) and Lonner et al. (2017). The specific measurement methods are presented in Figures 2, 3.

### Univariate Analyses

Continuous and categorical data were shown as mean ± standard deviation and numbers with percentages in parentheses, respectively. Shapiro-Wilk test was performed to test the normality of data distribution. Two-sided Student *t-*test (for normally distributed data) and Mann–Whitney–Wilcoxon test (for non-parametric data) were used to determine the statistical differences in continuous data between PJK and non-PJK groups, whereas chi-square test was performed for categorical variables. *p* < 0.1 was indicative of a statistically significant difference.

### Machine Learning Model Construction

Thirty-seven variables were normalized to reduce the effect of data scale while maintaining the distributions of original data. Data were split into training and testing sets at a random stratified ratio of 3:1 by preserving the percentage of samples for each class, and the testing set was held out for examining the generalization ability of the models. To address the class imbalance problem which could lead to a severely imbalanced degree of accuracy with the majority class having nearly 100% accuracy while the minority one having worse accuracy of 0–10%, two oversampling methods, the synthetic minority technique (SMOTE) (Chawla et al., 2002) and random oversampling (ROS) (He and Garcia, 2009), were applied for model training (Mendoza-Lattes et al., 2011; Lei et al., 2016, 2017; Lan, 2017; Sebaaly et al., 2018).

We established four kinds of popular supervised machine learning models [random forest (RF), support vector machine (SVM), k neighbors classifier (KNN), and linear regression (LR)] for risk prediction, which had superior advantages in solving the small-sample size problem. The parameters of the model were optimized by cross-validated grid search over a parameter grid, such as the number of estimators and criterion and the minimum number of samples required to split for RF; kernel, regularization parameter, and gamma for SVM; and number of neighboring samples, power parameter for the Minkowski metric, and weight function for KNN (Swami and Jain, 2012; Peng et al., 2016; Zhao et al., 2019). Leave-one-out cross-validation was implemented to evaluate the performance of models in training stage. More specifically, one patient from all patients was used for model testing while the rest for training, and these procedures were repeated until each patient had been used once as a testing sample. Final evaluation was be done with the independent test set as the model training was fulfilled to reflect the ability of a model to unknown sample.

Model discrimination was measured by area under the receiver operating characteristic curve (AUC). Accuracy was used to assess the difference between the predicted clinical results (PJK) and ground truth derived from follow-up study. The clinical utility of the model was also evaluated with F1 score, which is a necessary synthesized indicator by conveying the balance between the precision and the recall in imbalanced dataset (Chawla et al., 2002). At last, the model with the best prognostic performance was considered as the final prediction model to obtain the feature importance in PJK occurrence by ranking factor influences (Ji et al., 2015). Python version 3.5 (Python Software Foundation, Wilmington, DE, United States) was used for modeling.

### Cox Proportional Hazards Regression

A Cox proportional hazards regression model was also applied to select PJK-related features. Event-free survival was defined as the time from the date of surgery to the date of PJK occurrence. Follow-up time for patients without complications were censored at the last visit, and PJK patients contributed follow-up time until the outcomes were first recorded. The predictors of PJK with statistical significance in the univariable analysis were included in the multivariable Cox model. The final model was selected by forward Wald method. And the proportional hazards assumption of models was verified by examining the scaled Schoenfeld residual plots. The results were compared with the feature importance information acquired by machine learning model for exploring the interpretability and predictive value of variables. Statistical analysis was performed using SPSS 25.0 (IBM Corp., Armonk, NY).

## Results

### Clinical Characteristics

Tables 1, 2 show detailed baseline and clinical-radiologic characteristics of all patients. A total of 44 patients (female/male: 34/10) were recruited for this study. The average age at surgery, follow-up time, and instrumented vertebras were 18.27 ± 3.61 years, 3.15 ± 2.67 years, 6.80 ± 1.37 vertebras, respectively. At final follow-up, there were 10 (22.7%) patients with PJK, while 34 patients demonstrated no significant PJK by follow-up investigation.

**TABLE 1:** Demographic and clinical variables.

**Variable**	**None (*n* = 34)**	**PJK (*n* = 10)**	***p-*value**
Age at surgery, mean ± SD	18.50 ± 3.71	17.50 ± 3.31	0.669
Gender Female, ***n*** (%) Male, ***n*** (%)	30 (68.2%) 4 (9.1%)	4 (9.1%) 6 (13.6%)	0.001* (χ ^2^ = 10.23)
BMI	18.84 ± 2.39	21.29 ± 6.31	0.216
Amount of correction	80.4% ± 14.5%	81.6% ± 12.6%	0.901
Follow-up time (years)	2.88 ± 1.32	4.22 ± 4.54	0.648
UIV levels T1–T5 T6–T9 T10–T12	6 (13.6%) 20 (45.5%) 8 (18.2%)	2 (4.5%) 8 (18.2%) 0	0.232 (*c*^2^ = 2.92)
LIV levels L3 L4 L5	6 (13.6%) 18 (40.9%) 10 (22.7%)	5 (11.4%) 3 (6.9%) 2 (4.5%)	0.114 (χ^2^ = 4.338)
Number of instrumented vertebrae, mean ± SD	6.85 ± 1.31	6.60 ± 1.075	0.344

Bold and * values both represent a statistically significant difference between the PJK and None groups. SD, mean ± standard deviations; BMI, body mass index; UIV, upper instrumented vertebra; LIV, lower instrumented vertebra.

**TABLE 2:** Radiographic variables.

**Abbreviation**	**Parameter**	**Type**	**None (*n* = 34)**	**PJK (*n* = 10)**	***p*-value**
Coronal parameters					
CAMSC	The coronal main scoliosis curve Cobb angle (°)	Pre Post	43.96 ± 10.60 8.56 ± 6.31	39.74 ± 5.91 7.01 ± 4.81	0.237 0.478
CVA	Coronal vertical axis (mm)	Pre Post	14.01 ± 14.42 5.89 ± 18.86	16.69 ± 18.00 8.57 ± 12.18	0.628 0.675
Sagittal parameters					
TK	Thoracic kyphosis (°)	Pre Post	21.45 ± 9.42 19.35 ± 8.66	29.51 ± 11.68 23.50 ± 10.07	0.030* 0.207
LL	Lumber lordosis (°)	Pre Post	47.67 ± 11.43 48.54 ± 9.11	52.64 ± 10.20 47.20 ± 7.61	0.772 0.673
ULL	Upper segmental lordosis from L1 to L4 (°)	Pre Post	19.13 ± 9.19 20.55 ± 5.84	20.60 ± 934 19.20 ± 7.16	0.660 0.544
LLL	Lower segmental lordosis from L4 to S1 (°)	Pre Post	34.88 ± 8.84 31.23 ± 8.31	39.11 ± 10.97 30.11 ± 7.34	0.215 0.704
SVA	Sagittal vertical axis (°)	Pre Post	−8.77 ± 27.36 3.13 ± 30.83	1.39 ± 15.81 10.85 ± 25.78	0.271 0.476
PT	Pelvic tilt (°)	Pre Post	7.23 ± 7.84 3.68 ± 8.43	4.96 ± 10.03 3.31 ± 9.14	0.452 0.905
PI	Pelvic incidence (°)	Pre	45.12 ± 11.48	42.30 ± 13.61	0.514
SS	Sacral slope (°)	Pre Post	37.89 ± 8.73 39.70 ± 8.23	37.34 ± 5.87 37.72 ± 5.80	1.000 0.483
PI-LL mismatch	Pelvic incidence-lumbar lordosis mismatch (°)	Pre Post	−3.92 ± 12.69 −2.88 ± 9.30	10.35 ± 14.77 −6.10 ± 13.89	0.196 0.397
T1S	T1 slope (°)	Pre Post	14.43 ± 7.50 12.33 ± 7.93	19.45 ± 8.47 16.93 ± 5.82	0.078* 0.097*
T1SPI	T1SpinoPelvic inclination (°)	Pre Post	−4.07 ± 3.37 −1.87 ± 4.19	−3.05 ± 2.57 −1.03 ± 3.47	0.383 0.569
PJA	(°)	Pre Post	8.23 ± 5.45 7.74 ± 5.23	8.89 ± 3.64 13.35 ± 4.28	0.745 0.003*
UIV – UIV + 1	(°)	Pre Post	4.93 ± 3.47 4.45 ± 3.12	6.63 ± 2.97 8.13 ± 3.38	0.115 0.001*
RCA	Rod contour angle (°)	Post	4.03 ± 2.60	5.71 ± 4.56	0.464

All values are shown as mean ± SD. *Values represent a statistically significant difference (p < 0.1) between the PJK and None groups. Pre, preoperative; Post, immediate postoperative (3–7 days after surgery); UIV, upper instrumented vertebrae.

Between PJK and non-PJK groups, significant differences (*p* < 0.1) were observed in the following variables: gender distribution (*p* = 0.001), preoperative TK (*p* = 0.03), preoperative T1S (*p* = 0.078), postoperative T1S (*p* = 0.097), PJA (*p* = 0.003), and postoperative UIV – UIV + 1 (*p* = 0.001). However, there were no differences in age at surgery, body mass index (BMI), amount of correction, and UIV and LIV levels (Table 1). Additionally, no significant differences were observed in preoperative data including CAMSC, CVA, LL, ULL, LLL, SVA, PT, PI, SS, PI-LL mismatch, T1SPI, PJA, and UIV – UIV + 1, and immediate postoperative parameters including CAMSC, CVA, TK, LL, ULL, LLL, SVA, PT, PI, SS, PI-LL mismatch, T1SPI, and RCA (Table 2).

### Machine Learning Results

The average accuracies of machine learning models without oversampling for predicting PJK occurrence in the train and test sets were 0.728 and 0.783, whereas, models trained with ROS were 0.80 and 0.73, and models with SMOTE were 0.82 and 0.78, respectively. The average AUC for models without oversampling, with ROS, and with SMOTE were 0.64, 0.86, and 0.82 in the train set, respectively, and 0.70, 0.74, and 0.78 in the test set. The F1 score performances of the models that trained with oversampling were superior to that of without oversampling in both sets (train: 0.70 vs. 0.38, 0.70 vs. 0.24; test: 0.37 vs. 0.24, 0.53 vs. 0.24). The general tendency was that models with data oversampling had better robustness than the ones without preprocessing, and models that integrated SMOTE in the training stage yielded the best prognostic performance.

**FIGURE 4:**
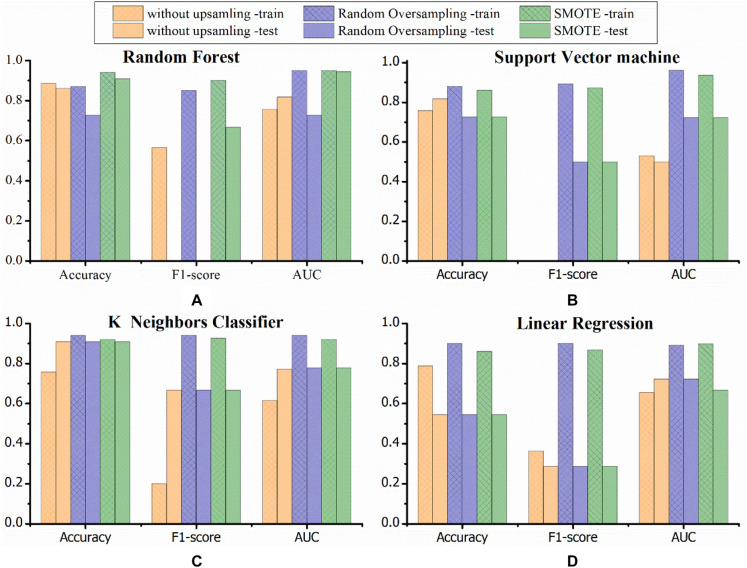
Graphs show the performances for PJK risk prediction obtained by established models in the training and testing sets. Three colors demonstrate different data processing methods (orange, without data processing; blue, random oversampling; green, SMOTE). Random forest combined with SMOTE provided an excellent prediction performance compared with rival models. SMOTE, the synthetic minority technique; AUC, area under the receiver operating characteristic curve; PJK, proximal junctional kyphosis. **(A–D)** Respectively represent the model performance of random forest, support vector machine, K neighbors classifier, linear regression.

**FIGURE 5:**
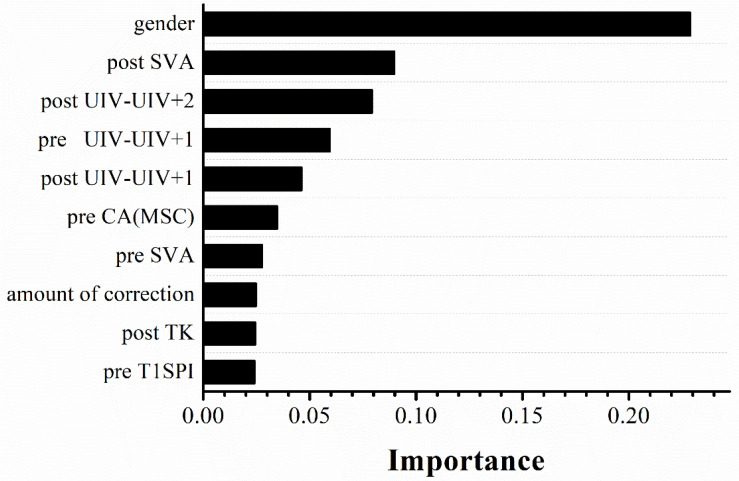
Importance order of top 10 predictors (importance = 64%) ranked by random forest using SMOTE. SMOTE, the synthetic minority technique; pre, pre-operation; post, immediate postoperative (3–7 days after surgery); SVA, sagittal vertical axis; UIV, upper instrumented vertebrae; CA(MSC), the main scoliosis curve coronal angle; TK, thoracic kyphosis; T1SPI, T1SpinoPelvic inclination.

Discriminatory performance and prediction accuracy of all models in leave-one-out cross-validation and test set are shown in Figure 4. Random forest using SMOTE provided better prognostic ability (AUC = 0.944), better clinical usefulness compared with rival models (accuracy = 0.909, F1 score = 0.667), and low operation time (4 ms for each sample) in independent test set, whereas, linear regression had the worst performance (AUC = 0.545, F1 score = 0.228, accuracy = 0.704), suggesting non-linear machine learning models had more precise prognostication. The detailed prediction outputs of this model were nine true negative, one false negative, one true positive, and zero false positive on test data set, demonstrating a lower misdiagnosis rate. In addition, the model presented feature selection based on data attributes importance ranking, and the top 10 prognostic indicators were gender, four preoperative features (UIV – UIV + 1, CAMSC, SVA, and T1SPI), and five modifiable surgical features (SVA, PJA, UIV – UIV + 1, TK, and amount of correction) (Figure 5).

### Multivariable Proportional Hazards Regression Model

To compare the feature selection results with the risk factors of PJK obtained by a model widely used for clinical research, a Cox proportional hazards regression model was also used. There were no significant violations of the proportional hazards assumption assessed by Schoenfeld residuals against time for all six statistically significant variables at univariable analysis. Multivariable Cox model based on aforementioned parameters demonstrated that male gender and larger preoperative T1S were the independent risk factors [odds ratio (OR) = 10.701 and 57.074, respectively] in Table 3. Gender was at the first place on the importance ranking in RF model, which accounted for 22.9%, compared with 1.2% of preoperative T1S.

**TABLE 3:** Cox proportional hazards regression model (forward Wald method) for risk factors of PJK.

**Variable**	**B**	**SE**	**Wald**	**df**	**Sig.**	**Exp(B)**	**95%CI for Exp(B)**	
							**Lower**	**Upper**
Male gender	2.370	0.719	8.804	1	0.002	10.701	2.062	34.510
Preoperative T1S	4.044	0.753	9.909	1	0.022	57.074	2.446	46.813

*T1S, T1 slope.*

## Discussion

The aim of our study was to develop prognostic models in Lenke 5 AIS patients undergoing long posterior instrumentation and fusion surgery and simultaneously explore the predictive value of clinical factors for PJK. We concluded that random forest that trained with SMOTE exhibited better performance in PJK prediction compared with other models. Specifically, in independent test set, the model provided better prognostic ability (AUC = 0.944, accuracy = 0.909, F1 score = 0.667) compared with other rival models, suggesting the reproducibility and reliability of the proposed model. In addition, a multivariable Cox model revealed that male gender and larger preoperative T1S were the independent prognostic factors for PJK (OR of male gender, 10.701 and OR of preoperative T1S, 57.074), and gender also ranked the first place with the prognostic importance of 22.9% in our prediction model.

For AIS patients, PJK was a complication after corrective surgery with unknown causation, and 22.7% of the patients in our study developed PJK (Hollenbeck et al., 2008; Zhao et al., 2018). The occurrence of PJK is multifactorial, including clinical, surgical, and radiographic factors. Linear regressions, such as binary logistic regression, may be simple and transparent for data analysis, however, they are not able to meet the needs of distinguishing high-dimensional and linear inseparable input data. Conversely, the power and potential of machine learning are increasingly recognized in the field of scoliosis correction (Group et al., 2015). In our study, we established four classes of models for PJK prediction. Models trained with oversampling methods showed relatively higher discrimination ability than that without using oversampling, suggesting rebalancing the class distribution for an imbalanced dataset was favorable to the construction of classifiers. In fact, SMOTE oversamples minority class by creating “synthetic” examples to build larger decision regions that contain nearby minority class points, rather than by oversampling with replacement, which actually diminishes and specifies the decision region for the minority class (Chawla et al., 2002; Ji et al., 2016). Our results also showed that random forest using SMOTE would be a useful approach that could effectively evaluate the risk of PJK postoperatively for patients with scoliosis in real time. In addition, the models may facilitate individualized surveillance policy. Specifically, low-risk patients may receive a less intensive surveillance regimen, even within the first year after surgery.

We carefully considered the potential risk factors for PJK. Several disputable factors were controlled in our study, including age, gender, TK, postoperative PJA, and UIV location. For example, UIV located in the lower thoracic region is a risk factor for PJK in Zhao et al. (2018), however, Zhao et al. recruited more PJK patients corrected by selected fusion with UIV stopping at lower thoracic levels, whereas, UIV always tended to stop at the upper thoracic regions (upper/lower: 36/8) in our study, which decreased the risk of PJK. In addition, we also included cervical alignment parameters in the analysis. T1S and male gender were independent risk factors in the multivariable Cox model when adjusting for other clinical prognostic factors. In fact, researchers have found that if middle or upper thoracic segments were fused, the postoperative compensation of cervical curvature would occur during the follow-up period (Sebaaly et al., 2018; Alzakri et al., 2019; Buell et al., 2019). We inferred that the proximal kyphosis might aggravate in PJK group to balance the cervical curvature for maintaining the global balance. Controversy exists on whether gender has an effect on the incidence of PJK or not. In accordance with Kim et al. (2007), which retrospectively assessed 410 patients and demonstrated that male gender had higher prevalence than female gender, our findings also suggested that male gender correlated significantly with PJK, although the underlying reasons were unclear.

Even though there were no differences in other sagittal spinopelvic parameters in Cox regression analysis, their importance in compensating for the misalignment of the spine in the long-term follow-up could not be ignored. In fact, the random forest model demonstrated that the top 10 prognostic indicators were gender, four preoperative features (UIV – UIV + 1, CAMSC, SVA, T1SPI), and five modifiable surgical parameters (SVA, PJA, UIV – UIV + 1, TK, amount of correction). Accordingly, the common points and differences between the results of the Cox model and the feature ranking extracted by the random forest model certified the significance of combined use of machine learning and statistical analysis. Five modifiable parameters of the prediction model may further supply a detailed assistant decision-making for preoperative surgical plan. We believe that our prediction models would affect operational design by individualizing management according to the risk profiles for PJK occurrence.

Our study had limitations. First, we developed our model for the Lenke 5 AIS patients, the most common Lenke type (Yang, 2003). However, further validation studies are warranted for other scoliosis types. Second, it was a retrospective analysis that suffers from inherent biases, although an independent data set was conducted to improve the reliability. Third, the sample size of this study was relatively small; our results require further validation with other institutions to check for the generalizability.

## Conclusion

In conclusion, the random forest using SMOTE model has great value for predicting the individual risk of developing PJK after long instrumentation and fusion surgery in Lenke 5 AIS patients. The model may facilitate clinical decision making in the era of precision medicine for spinal orthopedics. The combination of the results of a Cox model and the feature ranking extracted by machine learning is a promising approach to identify prognostic factors and has great significance in the medical field. Further studies are required to explore the generalized utility of our model and translate the results into clinical practice.

## Data Availability Statement

The datasets presented in this article are not readily available because, the datasets generated during and/or analyzed during the current study are available from the corresponding author on reasonable request. Requests to access the datasets should be directed to LP, pengli_bonne@163.com.

## Author Contributions

LP, XZ, and GZ conceived and launched this study. XiaoY and YG designed the medical and statistical analysis. YS, PX, BH, and XiY collected cases and clinical diagnosis. LP and RY took the angle measurements in X-rays. LP and LL analyzed the data, carried out statistical experiments, and wrote the first draft of this manuscript. LL and XZ revised and edited the final version. All authors reviewed and approved the manuscript.

## Conflict of Interest

The authors declare that the research was conducted in the absence of any commercial or financial relationships that could be construed as a potential conflict of interest.
