# Supplementary material for: Prediction of Proximal Junctional Kyphosis After Posterior Scoliosis Surgery With Machine Learning in the Lenke 5 Adolescent Idiopathic Scoliosis Patient
Source: Front Bioeng Biotechnol. 2020 Oct 6;8:559387. doi: 10.3389/fbioe.2020.559387 (PMC7573316; doi:10.3389/fbioe.2020.559387)
Supplement: Supplementary file 1 [file Data_Sheet_1.PDF]

Supplementary TABLE 1 | Performances for PJK risk prediction obtained from established models in the training stage by Leave-one-out cross-validation.

Model \ Oversampling	Without oversampling			RO			SMOTE		
	Accuracy	F1 score	AUC	Accuracy	F1 score	AUC	Accuracy	F1 score	AUC
RF	0.886	0.565	0.756	0.870	0.850	0.950	0.940	0.900	0.950
SVM	0.759	0	0.53	0.880	0.893	0.932	0.860	0.873	0.936
KNN	0.758	0.2	0.615	0.940	0.941	0.940	0.920	0.926	0.920
LR	0.788	0.363	0.655	0.900	0.900	0.891	0.860	0.898	0.899

RO, random oversampling; SMOTE, the synthetic minority technique; RF, random forest; SVM, support vector machine; KNN, k neighbors classifier; LR, linear regression.

Supplementary TABLE 2 | Performances for PJK risk prediction obtained from established models in the training stage by 3-fold cross-validation.

Model \ Oversampling	Without oversampling			RO			SMOTE		
	Accuracy	F1 score	AUC	Accuracy	F1 score	AUC	Accuracy	F1 score	AUC
RF	0.760	0.20	0.520	0.820	0.850	0.970	0.900	0.851	0.950
SVM	0.606	0.298	0.685	0.840	0.869	0.936	0.840	0.865	0.926
KNN	0.788	0.348	0.776	0.788	0.348	0.776	0.788	0.348	0.776
LR	0.759	0.685	0.590	0.757	0.756	0.869	0.741	0.735	0.842

RO, random oversampling; SMOTE, the synthetic minority technique; RF, random forest; SVM, support vector machine; KNN, k neighbors classifier; LR, linear regression.

Supplementary TABLE 3 | Performances for PJK risk prediction obtained from established models by Leave-one-out cross-validation. Data were split into training and testing sets at a random stratified ratio of 3:2.

Model \ Oversampling		Without oversampling			RO			SMOTE		
		Accuracy	F1 score	AUC	Accuracy	F1 score	AUC	Accuracy	F1 score	AUC
RF	Train	0.823	0.801	0.856	0.898	0.861	0.901	0.912	0.899	0.930
	Test	**0.778**	**0**	**0.339**	**0.833**	**0.400**	**0.286**	**0.778**	**0.333**	**0.339**
SVM	Train	0.769	0	0.775	0.900	0.900	0.9375	0.775	0.781	0.900
	Test	**0.778**	**0**	**0.535**	**0.778**	**0.333**	**0.607**	**0.833**	**0.571**	**0.607**
KNN	Train	0.654	0.308	0.541	0.825	0.844	0.983	0.775	0.781	0.900
	Test	**0.778**	**0.5**	**0.678**	**0.611**	**0.363**	**0.589**	**0.833**	**0.571**	**0.607**
LR	Train	0.808	0.286	0.767	0.875	0.878	0.96	0.850	0.857	0.977
	Test	**0.833**	**0.4**	**0.5**	**0.667**	**0.25**	**0.554**	**0.722**	**0.445**	**0.536**

RO, random oversampling; SMOTE, the synthetic minority technique; RF, random forest; SVM, support vector machine; KNN, k neighbors classifier; LR, linear regression.

Supplementary TABLE 4 | Performances for PJK risk prediction obtained from established models by Leave-one-out cross-validation. Data were split into training and testing sets at a random stratified ratio of 1:1.

Model \ Oversampling		Without oversampling			RO			SMOTE		
		Accuracy	F1 score	AUC	Accuracy	F1 score	AUC	Accuracy	F1 score	AUC
RF	Train	0.85	0.578	0.8	0.878	0.686	0.875	0.88	0.726	0.899
	Test	**0.818**	**0.333**	**0.541**	**0.772**	**0.285**	**0.565**	**0.818**	**0.333**	**0.553**
SVM	Train	0.772	0	0.612	0.912	0.919	0.966	0.912	0.919	0.934
	Test	**0.772**	**0**	**0.648**	**0.681**	**0.461**	**0.694**	**0.681**	**0.461**	**0.694**
KNN	Train	0.772	0	0.471	0.882	0.889	0.882	0.882	0.894	0.882
	Test	**0.772**	**0**	**0.805**	**0.591**	**0.308**	**0.524**	**0.591**	**0.4**	**0.594**
LR	Train	0.772	0	0.635	0.824	0.842	0.958	0.882	0.882	0.955
	Test	**0.818**	**0.5**	**0.659**	**0.636**	**0.429**	**0.682**	**0.682**	**0.462**	**0.671**

RO, random oversampling; SMOTE, the synthetic minority technique; RF, random forest; SVM, support vector machine; KNN, k neighbors classifier; LR, linear regression.
